# Biomarkers of Maternal and Neonatal Oxidative and Nitrosative Stress During Delivery: An Exploratory Pilot Study on Maternal–Neonatal Adaptation to Stress

**DOI:** 10.3390/jcm15156061

**Published:** 2026-08-04

**Authors:** Zsuzsánna Simon-Szabó, Sándor Pál, Enikő Nemes-Nagy, Lóránd Dénes, Mircea Dumitru Croitoru, Margit Solymár, Zsuzsanna Faust, Manuela Cucerea, Ana Maria Fárr, Hajnal Finta, Erzsébet Májai

**Affiliations:** 1 Pathophysiology Department, George Emil Palade University of Medicine, Pharmacy, Science and Technology of Târgu Mureș, 540142 Târgu Mureș, Romania; zsuzsanna.simon-szabo@umfst.ro (Z.S.-S.);; 2 Department of Neonatology, Mureș County Clinical Hospital, 540142 Târgu Mureș, Romania; 3 Department of Laboratory Medicine, Department of Transfusion Medicine, Medical School, University of Pécs, 7622 Pécs, Hungary; pal.sandor@pte.hu (S.P.); faust.zsuzsanna@pte.hu (Z.F.); 4 Chemistry and Medical Biochemistry Department, George Emil Palade University of Medicine, Pharmacy, Science and Technology of Târgu Mureș, 540142 Târgu Mureș, Romania; 5 Central Laboratory, Emergency Clinical County Hospital of Târgu Mureș, 540136 Târgu Mureș, Romania; 6 Anatomy and Embryology Department, George Emil Palade University of Medicine, Pharmacy, Science and Technology of Târgu Mureș, 540142 Târgu Mureș, Romania; 7 Laboratory of Radiology and Medical Imaging, Mureș County Clinical Hospital, 540072 Târgu Mureș, Romania; 8 Toxicology and Biopharmacy Department, George Emil Palade University of Medicine, Pharmacy, Science and Technology of Târgu Mureș, 540142 Târgu Mureș, Romaniaerzsebet.fogarasi@umfst.ro (E.M.); 9 Neonatology Department, George Emil Palade University of Medicine, Pharmacy, Science and Technology of Târgu Mureș, 540136 Târgu Mureș, Romania; manuela.cucerea@umfst.ro; 10 Neonatology Clinic, Emergency Clinical County Hospital of Târgu Mureș, 540136 Târgu Mures, Romania; 11 Department of Public Health and Health Management, George Emil Palade University of Medicine, Pharmacy, Science and Technology of Târgu Mureș, 540142 Târgu Mureș, Romania; 12 Department of Medical Evaluation and Statistics, Emergency Clinical County Hospital of Târgu Mureș, 540136 Târgu Mureș, Romania

**Keywords:** oxidative stress, nitrosative stress, maternal–neonatal dyads, delivery, cesarean section, Apgar score, redox biomarkers, maternal smoking

## Abstract

**Background/Objectives**: Perinatal oxidative and nitrosative stress may contribute to maternal–neonatal adaptation during delivery. This pilot study evaluated pathway-specific oxidative and nitrosative stress markers from blood samples in paired mother–newborn dyads and explored their associations with delivery-related variables, maternal smoking habits and neonatal clinical state. **Methods**: The study included 74 mother–newborn dyads, yielding 148 paired maternal and neonatal investigations. Biomarkers included plasma nitrite, nitrate, malondialdehyde, and reduced and oxidized glutathione. Composite pathway-specific indices were calculated: an Oxidative Stress Index (OSI) and a Nitrosative Stress Index (NSI). **Results**: Paired testing showed that OSI was significantly higher in the maternal compartment compared to the neonatal compartment (*p* < 0.001), while NSI showed no significant difference (*p* = 0.594). Maternal and neonatal indices showed positive monotonic correlations (OSI Spearman’s rho = 0.608, *p* < 0.001; NSI Spearman’s rho = 0.275, *p* = 0.018). Following false discovery-rate correction (FDR) across the exploratory inferential analysis, no raw or composite biomarkers showed statistically significant differences. **Conclusions**: This study indicates that while maternal and neonatal oxidative indices are monotonically correlated, their absolute concentrations shift significantly across compartments, highlighting the need to treat these indices as strictly exploratory, hypothesis-generating metrics.

## 1. Introduction

Labor and childbirth are accompanied by substantial physiological and biochemical changes, including alterations in redox balance. During labor, myometrial contractions may periodically reduce uteroplacental blood flow, producing intermittent ischemia–reperfusion episodes across the placental bed [1]. These processes can activate stress-signaling pathways, including Nuclear Factor Kappa B (NF-κB), Mitogen-Activated Protein Kinase 38 (p38 MAPK), and Stress-Activated Protein Kinase/c-Jun N-terminal Kinase (SAPK/JNK) pathways, and may contribute to the release of proinflammatory cytokines, prostaglandins, and apoptotic mediators in a duration-dependent manner [2,3]. Increased mitochondrial oxygen consumption during labor may also enhance electron leakage from the respiratory chain, contributing to the generation of reactive oxygen species (ROS), including superoxide radicals, hydrogen peroxide, and hydroxyl radicals [4,5]. The placenta itself is an important site of ROS and reactive nitrogen species (RNS) production through several enzymatic and mitochondrial pathways, including Nicotinamide Adenine Dinucleotide Phosphate (NADPH) oxidase, xanthine oxidase, and the mitochondrial electron transport chain [5].

Reactive nitrogen species also appear to be relevant to parturition physiology. Nitric oxide (NO), produced by endothelial NO synthase, is involved in the regulation of fetoplacental vascular tone, vasodilatation, antithrombotic activity, and angiogenesis [5,6]. Nitrite, previously regarded mainly as an end-product of NO oxidation, may act as an NO reservoir in placental tissue, particularly under hypoxic conditions through the Nitric oxide Soluble Guanylyl Cyclase Cyclic Guanosine Monophosphate (NO-sGC-cGMP) pathway [7]. Nitrite may also participate in the formation of iron nitrosyl complexes in placental tissue, potentially affecting iron-containing proteins involved in mitochondrial function and regulatory processes [8,9].

At birth, the neonate undergoes an abrupt transition from the relatively hypoxic intrauterine environment to the normoxic extrauterine environment. This increase in oxygen concentration can contribute to mitochondrial ROS generation [10,11]. Newborns are particularly susceptible to oxidative stress because antioxidant defenses are still developing, and levels of several antioxidants and metal-binding proteins are relatively limited [11,12]. During late gestation, the fetal antioxidant system undergoes important maturation, including increasing transfer of nonenzymatic antioxidants and upregulation of endogenous antioxidant enzymes [13]. Term labor may also be associated with compensatory changes in fetal antioxidant reserve, which could help the neonate adapt to the oxidative challenges of birth [14].

Perinatal redox research has often relied on individual biomarkers, such as MDA for lipid peroxidation, GSH for antioxidant reserve, and nitrite or nitrate for nitric oxide metabolism [15,16,17]. Although such biomarkers are informative, their isolated interpretation has limitations. Circulating biomarkers may reflect generalized oxidative status, may not identify tissue source, and may lack sufficient sensitivity or specificity when interpreted alone [18]. No single biomarker has been validated as a definitive measure of redox status in clinical samples [19]. Multimarker approaches, including latent-variable and composite index strategies, have been proposed to reduce biomarker-specific variability and provide a broader estimate of oxidative stress or redox imbalance [20,21]. In small clinical cohorts, reducing the number of separate biomarker endpoints may also help limit type I error inflation and overfitting.

Another important concern is to what extent the redox states of the mother and newborn are linked via the placenta. A simple passive diffusion model would imply a close maternal–neonatal equilibration of oxidative and nitrosative markers. However, the placenta is a metabolically active organ with its own ROS/RNS-producing capacity and antioxidant defense systems, including glutathione-related enzymes concentrated in placental and decidual tissues [22,23]. The glutathione-dependent detoxification system appears to function locally within placental tissue and may not simply shuttle reduced glutathione between maternal and fetal compartments [23,24,25]. These findings suggest that maternal and neonatal redox profiles may show partial compartmentalization or segregation rather than complete passive equilibration.

Mode of delivery may also influence neonatal redox adaptation. Vaginal delivery exposes the fetus to intermittent uterine-contraction-associated with hypoxic–ischemic and reperfusion-like episodes, which may act as a physiological preconditioning trigger [1,2]. Vaginal delivery is also associated with a sympathoadrenal catecholamine surge that contributes to lung fluid clearance, surfactant secretion, peripheral vascular regulation, and metabolic adaptation [26,27,28]. Cesarean section, particularly when performed without labor, may bypass some aspects of this gradual labor-associated adaptive response. Previous studies have reported differences in neonatal oxidative stress markers and antioxidant status according to delivery mode, although results vary depending on timing, study population, and biomarker selection [29,30,31,32].

Maternal tobacco smoke exposure may represent an additional chronic stressor affecting the fetal–placental redox environment. Tobacco smoke contains numerous compounds that can increase ROS generation and contribute to placental oxidative stress, lipid peroxidation, altered antioxidant capacity, and inflammatory pathway activation [33,34,35,36,37]. These mechanisms may reduce fetal–placental antioxidant reserve and could influence neonatal adaptation during delivery, although associations with neonatal clinical parameters require cautious interpretation, especially in small cohorts.

Based on these considerations, the present exploratory pilot study aimed to evaluate pathway-specific oxidative and nitrosative stress patterns in mother–newborn pairs. Specifically, we aimed to develop composite oxidative and nitrosative stress indices to reduce reliance on single biomarkers, and to evaluate the correlations between maternal–neonatal indices.

## 2. Materials and Methods

**Figure 1:**
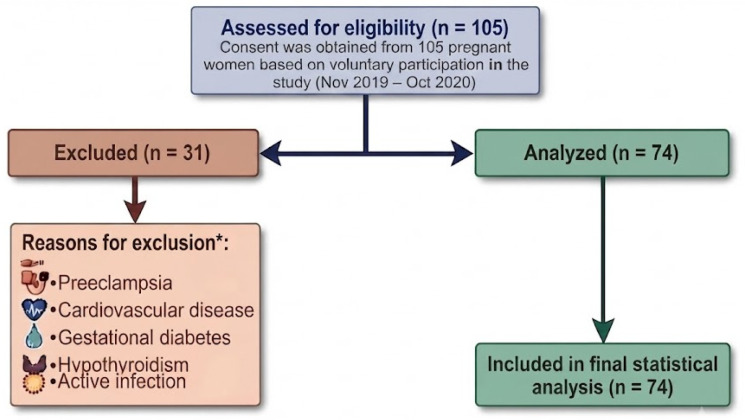
Participant enrollment flow. 105 pregnant women provided consent, of whom 31 were excluded based on pre-defined criteria, and the remaining 74 mother-newborn dyads were enrolled in the study. * indicates the pathologies on the basis of which cases were excluded from the study based on the predefined criteria.

The study cohort was recruited from the Obstetrics-Gynecology Clinic I of the Emergency County Clinical Hospital (ECCH) in Târgu Mureș between November 2019 and October 2020. Participation was voluntary. Ethical approval was granted by the Institutional Review Boards of both the ECCH and the George Emil Palade University of Medicine, Pharmacy, Science, and Technology of Târgu Mureș, Romania (GEP UMphST). Exclusion criteria included preeclampsia, pre-existing cardiovascular disease, gestational diabetes, hypothyroidism, multiple fetuses or the presence of an active infection at the time of admission. A total of 105 pregnant women initially provided informed consent and completed a self-report questionnaire covering personal information, biometric data, lifestyle and medical history. Following a primary assessment upon admission, 31 participants were excluded based on the predefined criteria. Consequently, the final study population comprised 74 mother–newborn pairs, yielding 148 total complex datasets, as shown in Figure 1.

The raw data included nitrite (NO_2_), nitrate (NO_3_), malondialdehyde (MDA), reduced glutathione (GSH), and oxidized glutathione (GSSG) determined from maternal venous blood sample and umbilical cord blood. The blood samples underwent centrifugation at 3000 rpm for 10 min and the obtained plasma samples were frozen at −80 °C until assessment via high-performance liquid chromatography (HPLC) method. Gestational age, birth weight, mode of delivery, Apgar scores, maternal smoking status, and oxytocin administration were also documented and analyzed. Maternal BMI and parity were not documented.

No formal prior sample size calculation was performed. This study was designed and is presented as an exploratory pilot investigation intended to generate hypotheses for larger, adequately powered confirmatory studies.

To obtain a continuous scale for gestational age, non-standard entries, such as slash- or dash-separated values, were transformed into their mean numeric equivalents. Mode of delivery was categorized as vaginal delivery or cesarean section. Maternal smoking was treated as a binary indicator. A unique dyad identifier (Dyad_ID) was generated to preserve the paired maternal–neonatal data structure.

The primary analytical system for all measurements was a Merck-Hitachi HPLC UV/VIS system (Merck KGaA, Darmstadt, Germany).

System Configuration: L-7100 quaternary pump, L-7200 injector/autosampler, L-7360 column thermostat, D-7000 interface, L-7612 solvent degasser, and L-7455 DAD detector.

Supplementary equipment:

◦ AB545 analytical balance (Mettler-Toledo, Greifensee, Switzerland).
◦ Centrifugation/Mixing: Centrifuge (Sigma-Aldrich, St. Gallen, Switzerland), Mix 20 vortex Falc Instruments, Treviglio, BG, Italy, T700H ultrasonic bath (Elma Schmidbauer GmbH, Singen, Germany).
◦ pH Measurement: MP 225 pH meter (Mettler-Toledo Quatro, Greifensee, Switzerland).
◦ Additional: Water purification system (Merck KGaA, Darmstadt, Germany).

### 2.1. Reagents and Materials

Merck KGaA (Darmstadt, Germany): All reagents for Nitrate/Nitrite measurement (including sulphanilic acid (p.a. ≥ 99.0%), 1-naphtylamine analytical standard, acetic acid 100%, sodium nitrite p.a., sodium nitrate p.a.), methanol gradient grade for HPLC, acetonitrile gradient grade for HPLC, phosphate buffer components (Na_2_HPO_4_, H_3_PO_4_), and general laboratory supplies.

Sigma-Aldrich (St. Gallen, Switzerland): thiobarbituric acid (TBA), 1,1,3,3-tetramethoxypropane (MDA standard), Ellman’s reagent (DTNB), and GSH/GSSG standards.

Chemical Company (Iași, Romania): Sulfuric acid and trichloroacetic acid.

### 2.2. Analytical Methodology and Data


**Parameter**

**Condition**

**Stationary Phase**
MDA determinationHPLC-UV/VIS methodSupelcosil LC-18 (3 µm)Nitrate/NitriteHPLC-UV/VIS ion-pair methodLichrospher 100 RP-18 (5 µm)GSH/GSSGHPLC-UV/VIS methodZorbax Eclipse XDB-C8 (5 µm)

- MDA measurement: Mobile phase: acetonitrile, phosphate buffer (20 mM, pH = 6), isocratic elution; detection wavelength 532 nm. Retention time: 0.58 min. Standard range: 0.52–262 ng/mL [38].
- Nitrate/Nitrite measurement: Mobile phase: tetrabutylammonium hydroxide 5 mM brought to pH 2.5 with sulfuric acid, acetonitrile and methanol, gradient elution; simultaneous detection via UV (222 nm for nitrate) and visible (520 nm for derivatized nitrite). Calibration ranges: nitrate (0.2–40 µg/mL), nitrite (10–400 ng/mL) [39].
- GSH/GSSG measurement: Mobile phase: acetonitrile, phosphate buffer (20 mM, pH = 2.5) gradient elution; detection wavelength 330 nm. Retention time: 9.11 min. Standard range: 0.5–100 µg/mL. GSSG determination involved heat-induced reduction at 80 °C for 30 min [40].

Detailed HPLC calibration, analytical standards and reagent preparation are presented as Supplementary Material in protocol P1.

All statistical analyses were performed using R version 4.5.3. The tidyverse suite (version 2.0.0) was used for data management, readxl (version 1.4.5) for data import, and gt (version 1.2.0) for table generation.

For statistical comparisons and correlation analyses, we utilized rstatix (version 0.7.2) to perform Wilcoxon signed-rank tests and Spearman rank correlations. Confidence intervals (95% CI) were computed via bootstrap resampling (2000 replicates) using the boot package (version 1.3-32).

Data visualization was accomplished primarily with ggplot2 (version 4.0.3), extended by ggpubr (version 0.6.0) for publication-ready formatting and ggExtra (version 0.11.0) for marginal plot distributions. The analytical sample was restricted to complete mother–newborn dyads. Descriptive summaries were calculated for the cohort and separately for maternal and neonatal compartments.

The overall redox framework was separated into two composite indices: the Oxidative Stress Index (OSI) and the Nitrosative Stress Index (NSI). These composite indices were newly created and were not externally validated in this population. The distinction was based on the biochemical separation between oxidative stress, represented by lipid peroxidation and glutathione turnover, and nitrosative stress, represented by nitric oxide metabolites. This approach was intended to reduce reliance on single biomarkers while preserving pathway-specific interpretation.

The separation of OSI and NSI is also consistent with the possibility that different biomarker classes may have different biological behavior across the maternal–neonatal interface. Lipid peroxidation products and glutathione-related molecules may not behave identically to smaller inorganic nitric oxide metabolites such as nitrite and nitrate. However, because these composite indices are not externally validated in this population, their interpretation should remain cautious.

Because the biomarker variables were non-negative and potentially right-skewed, MDA, NO_2_, NO_3_, GSH, and GSSG were log-transformed before standardization. Z-score standardization was then performed using the pooled maternal–neonatal distribution as the reference population [41,42].

$$Z_{\mathrm{Biomarker}}=\frac{log\left(\mathrm{Biomarker}\right)-\mu_{\mathrm{pooled}}}{\sigma_{\mathrm{pooled}}}$$

This pooled reference approach allowed maternal and neonatal values to be compared on a shared standardized scale.

### 2.3. Algebraic Sign Convention and Equal Weighting

Composite indices were calculated using an equal-weighting scheme.

OSI was calculated as

$$OSI=Z_{MDA}+Z_{GSSG}-Z_{GSH}$$

In this formulation, MDA and GSSG were treated as pro-oxidant or oxidative stress-associated markers, whereas GSH was treated as an antioxidant reserve marker. Therefore, higher OSI values were interpreted as indicating greater oxidative imbalance.

NSI was calculated as

$$NSI=Z_{NO2}+Z_{NO3}$$

Both components were entered with a positive sign because they represent nitric oxide-related metabolites.

An equal-weighting scheme was used because no validated differential weighting system was available for this specific perinatal population. This approach avoids deriving weights from the same dataset used for outcome modeling, which could increase the risk of overfitting.

Given the sample size of 74 mother–newborn dyads, conventional parametric and non-parametric statistical methods were used. The analyses were intended to be exploratory and hypothesis-generating.

Maternal–neonatal paired comparisons of OSI and NSI were performed using the Wilcoxon signed-rank test. This paired approach was used because each maternal observation was biologically linked to a corresponding neonatal observation.

The algebraic sign assigned to each biomarker reflects its presumed direction of biochemical effect rather than an empirically estimated weight. MDA and GSSG were assigned positive signs because both increase with pro-oxidative processes: MDA is a stable lipid peroxidation end-product, and GSSG represents the oxidized glutathione fraction generated during redox buffering. GSH was assigned a negative sign because it represents antioxidant reserve; higher GSH reflects greater buffering capacity and should lower, not raise, the oxidative imbalance index. Both nitrite and nitrate were assigned positive signs in NSI as downstream nitric oxide metabolites, without distinguishing protective vasoregulatory signaling from potentially harmful nitrosative modification, since the available biomarker panel did not permit this distinction.

Equal weighting (absolute weight = 1 for each standardized component) was a deliberate methodological choice rather than a default assumption. No externally validated reference redox score was available for this population against which alternative weightings could be benchmarked. Consequently, MDA, GSSG, GSH, nitrite, and nitrate were treated as biochemically equivalent contributors within their respective pathway, differing only in presumed direction of effect. Only nitrite and nitrate were included in NSI because these were the only nitrosative pathway biomarkers measured in this study; other nitrosative markers (e.g., 3-nitrotyrosine, S-nitrosothiols) were not assayed and their omission is acknowledged as a limitation of pathway coverage.

Maternal–neonatal correlations were evaluated using Spearman rank correlation. Correlations were calculated between maternal and neonatal OSI, and between maternal and neonatal NSI. Additionally, 95% confidence intervals (95% CI) were obtained from 2000 replicates of the sample using bootstrap resampling. The alpha threshold for statistical significance was set at 0.05 (two-sided) prior to multiplicity adjustment. Given the exploratory nature of this study, all interpretations are intended as hypothesis-generating.

## 3. Results

### 3.1. Cohort Characteristics

A total of 74 maternal–neonatal dyads were analyzed, comprising 55 (74.3%) vaginal deliveries and 19 (25.7%) cesarean deliveries. Overall, the median maternal age was 26.0 years (interquartile range [IQR]: 9.0 years; vaginal: 26.0 [IQR: 10.0] vs. cesarean: 28.0 [IQR: 7.5], *p* = 0.431), and the median gestational age at delivery was 38.5 weeks (IQR: 2.0 weeks; vaginal: 39.5 [IQR: 1.0] vs. cesarean: 38.5 [IQR: 5.8], *p* = 0.430). Intrapartum oxytocin administration was observed in 18 cases (24.3%; vaginal: 15/55 [27.3%] vs. cesarean: 3/19 [15.8%], *p* = 0.372), and maternal smoking during pregnancy was reported in 28 cases (37.8%; vaginal: 21/55 [38.2%] vs. cesarean: 7/19 [36.8%], *p* = 1.000). Among newborns, 42 (56.8%) were male (vaginal: 29/55 [52.7%] vs. cesarean: 13/19 [68.4%], *p* = 0.289). The median birth weight was 3160 g (IQR: 715 g; vaginal: 3250 g [IQR: 680] vs. cesarean: 3000 g [IQR: 1095], *p* = 0.151). Neonates born via vaginal delivery demonstrated significantly higher median Apgar scores at 1 min (9.0 [IQR: 1.5] vs. 8.0 [IQR: 2.5], *p* = 0.039) and at 5 min (10.0 [IQR: 1.0] vs. 9.0 [IQR: 2.0], *p* = 0.034) compared to those delivered via cesarean section. Baseline descriptive statistics stratified by maternal and neonatal compartments are presented in Table 1.

### 3.2. Maternal–Neonatal Differences in Redox Indices

Analysis of standardized pathway-specific indices showed different maternal–neonatal patterns for OSI and NSI. Paired Wilcoxon signed-rank testing showed a statistically significant maternal–neonatal difference in OSI, with neonates showing lower values than mothers (*p* = 0.0006). No significant difference was observed for NSI between compartments (*p* = 0.536).

Table 2 contains detailed data about the comparisons.

### 3.3. Maternal–Neonatal Correlation Structure

Spearman rank correlation analysis showed a weak and statistically non-significant correlation between maternal OSI and neonatal OSI (ρ = −0.128, *p* = 0.277). The correlation between maternal NSI and neonatal NSI was weak-to-moderate and statistically significant (ρ = 0.313, *p* = 0.007). Table 3 contains the data of the performed non-parametric (Spearman’s rho) correlation analyses.

**Figure 2:**
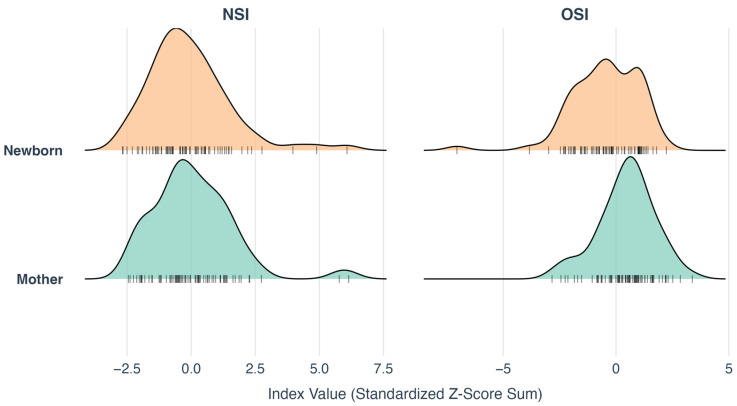
Density distributions of pathway-specific redox stress indices, OSI and NSI, across maternal and neonatal compartments. The plot displays smoothed kernel density estimates for each index. The upper row (light orange distributions) represents the newborn compartment, while the lower row (light green distributions) represents the maternal compartment. Solid black vertical tick marks along the baseline (rug plots) indicate individual standardized index values. Data points on the x-axis represent the standardized z-score sum, where negative values indicate levels below the population mean.

Figure 2 illustrates the density distributions of OSI and NSI across maternal and neonatal compartments, corresponding to the paired comparisons described above.

### 3.4. Exploratory Two-Dimensional Redox Phenotype Analysis

Exploratory two-dimensional quadrant analysis mapped OSI against NSI to visualize redox patterns across the cohort. The maternal compartment appeared to cluster mainly in regions with lower oxidative stress and variable nitrosative stress, whereas the neonatal compartment showed greater heterogeneity.

**Figure 3:**
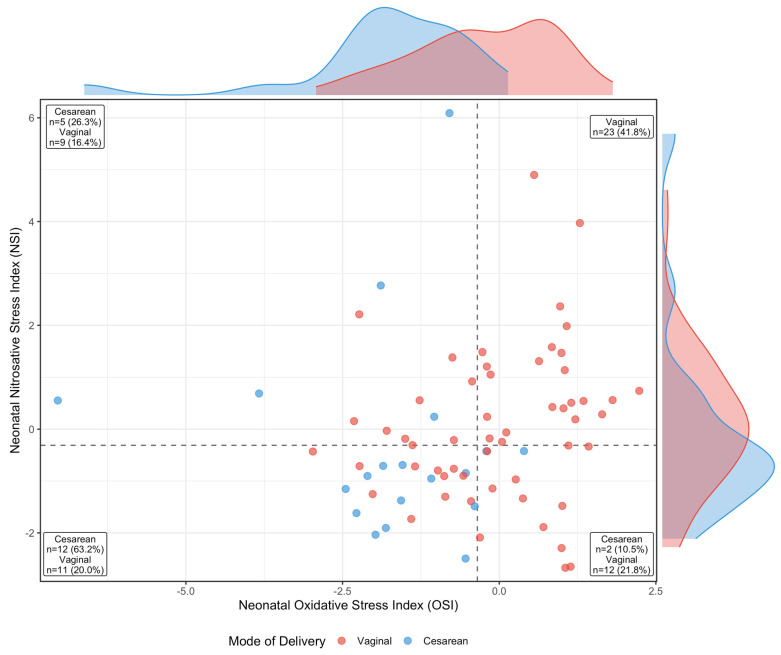
Quadrant mapping of neonatal Oxidative Stress Index (OSI) and Nitrosative Stress Index (NSI), stratified by mode of delivery (blue = cesarean, red = vaginal). Dashed lines indicate cohort median cutpoints; each corner label reports the number and percentage of neonates within that quadrant for each delivery mode. Marginal density curves along the top and right edges show the group-wise distribution of OSI and NSI, respectively, illustrating that vaginally delivered neonates were more frequently located in the high-OSI/high-NSI quadrant.

When stratified by mode of delivery, neonates delivered by cesarean section appeared visually more frequently in the low-OSI/low-NSI region. Figure 3 shows the exploratory two-dimensional quadrant mapping of neonatal OSI against NSI, stratified by mode of delivery, as described below.

## 4. Discussion

In this preliminary study of 74 mother–newborn dyads, pathway-specific oxidative and nitrosative stress indices showed differing maternal–neonatal patterns. OSI differed significantly between maternal and neonatal compartments, whereas NSI did not. Maternal–neonatal OSI correlation was weak and statistically non-significant, while NSI showed a weak-to-moderate statistically significant correlation. These findings suggest that oxidative and nitrosative pathways may not behave as a single unified redox construct and that maternal–neonatal redox relationships may show partial compartmentalization during delivery.

### 4.1. Maternal–Neonatal Redox Relationships and Possible Compartmentalization

The observed maternal–neonatal OSI difference, together with the weak OSI correlation, may challenge a simple passive-equilibration model of oxidative stress across the maternal–neonatal interface. The placenta is not merely a passive diffusion membrane; it has substantial metabolic activity, including ROS/RNS generation and antioxidant defense systems [5,22,23,24,25]. Therefore, weak maternal–neonatal coupling of OSI may be consistent with localized oxidative processes in maternal, placental, and neonatal compartments.

The neonatal transition from the intrauterine to extrauterine environment may also contribute to differences in oxidative stress patterns. At birth, the neonate experiences a marked increase in oxygen concentration, which may increase mitochondrial ROS generation [10,11,38]. At the same time, neonatal antioxidant systems are still maturing, and antioxidant reserve may vary according to gestational and perinatal factors [11,12,13,14]. These physiological conditions could contribute to differences in oxidative stress indices between maternal and neonatal samples.

The NSI pattern offered a different insight. Maternal and neonatal NSI did not differ significantly, and the maternal–neonatal NSI correlation was statistically significant but modest. These findings suggest that maternal and neonatal oxidative and nitrosative pathways do not fully equilibrate across the maternal–neonatal interface. The relatively weak OSI correlation and modest NSI correlation are consistent with partial compartmentalization, although these findings require confirmation in larger cohorts. This may reflect differences in the biological behavior of nitric oxide metabolites compared with lipid peroxidation and glutathione-related markers. Nitrite and nitrate are relatively stable end-products of nitric oxide metabolism and may have different transport or equilibration properties across the maternal–neonatal interface [7,8,9]. However, the modest magnitude of the correlation suggests that NSI also should not be interpreted as fully balanced between maternal and neonatal compartments.

### 4.2. Delivery Mode and Neonatal Redox Patterns

The exploratory quadrant analysis suggested that neonates delivered by cesarean section may be more frequently located in the low-OSI/low-NSI region. This visual pattern is biologically plausible but should be interpreted cautiously, particularly because it was descriptive and not independently confirmed.

Several mechanisms may contribute to delivery-mode-related redox differences. Vaginal delivery exposes the fetus to repeated uterine-contraction-associated changes in perfusion, which may act as a physiological preconditioning stimulus [1,2]. Labor is also associated with a catecholamine surge that supports neonatal cardiopulmonary and metabolic adaptation [26,27,28]. Cesarean section may reduce or bypass some of these labor-associated adaptive responses, depending on whether it is elective or performed after labor onset. Given the small number of cases, it was not possible to stratify cases of cesarean sections based on the presence or absence of labor or to conduct comparisons between emergency and elective cesarean sections; therefore, comparisons by subcategory could not be performed.

Prior studies have reported differences in cord blood oxidative stress and antioxidant markers according to delivery mode, but results are not fully consistent [29,30,31,32]. Differences between elective and emergency cesarean section, anesthesia, labor duration, maternal indications, and sampling timing may all influence redox measurements. Therefore, the present findings should be considered hypothesis-generating and require more detailed delivery-mode stratification in future studies. The clinical significance of these differences remains uncertain. Whether labor-associated oxidative stress represents adaptive physiological signaling that prepares the neonate for extrauterine life, or whether it contributes to subtle cellular damage, is not definitively established. The lower oxidative stress in elective cesarean may reflect loss of important developmental cues rather than a purely beneficial state.

### 4.3. Maternal Smoking and Neonatal Clinical Status

Tobacco smoke exposure may contribute to oxidative stress through increased ROS generation, lipid peroxidation, and reduced antioxidant capacity [33,34,35]. Smoking has also been associated with structural and molecular placental changes, including deoxyribonucleic acid (DNA) damage, telomere shortening, and altered expression of genes related to angiogenesis and apoptosis [36,37]. Smoking mothers represented 37.8% of the study participants; however, the present study was not designed to establish causality, and residual confounding cannot be excluded.

### 4.4. Strengths and Limitations

A strength of this study is the paired mother–newborn dyad design, which allowed direct evaluation of maternal–neonatal redox relationships using methods developed and validated by the authors to measure oxidative and nitrosative stress biomarkers [38,39].

The use of pathway-specific composite indices may also provide a broader view of oxidative and nitrosative stress than individual biomarkers alone.

Several limitations should be acknowledged. The sample size was limited, and the analyses were exploratory. The composite oxidative stress scores have been newly constructed in this manuscript, and are equally weighted, sample-dependent and the study relied on internal validation only; no external validation cohort was available. A specific limitation of the composite indices is that their sign convention encodes a directional biochemical hypothesis rather than an empirically validated relationship, and equal weighting assumes, without biological confirmation, that a one-SD change in each component contributes identically to the overall index. This approach was pragmatic and intended to avoid data-derived overfitting, but the weights have not been biologically or externally validated in this specific perinatal population. Due to a lack of data, residual confounding related to obstetric indications, labor duration, anesthesia, maternal comorbidities, or other perinatal factors may have gone undetected. Furthermore, the small sample size limits the generalizability of the findings beyond the present cohort, while also restricting stratification possibilities and comparisons between subcategories.

Future studies should evaluate and improve through development these indices in larger, preferably multicenter cohorts. Such studies should include more detailed stratification by mode of delivery, labor exposure, obstetric indication, smoking intensity, and neonatal clinical outcomes.

## 5. Conclusions

This preliminary study suggests that pathway-specific oxidative and nitrosative stress indices may provide additional information on maternal–neonatal redox relationships during delivery. The weak OSI correlation and modest NSI correlation between maternal and neonatal compartments are consistent with partial compartmentalization rather than complete passive equilibration. Exploratory analyses suggested possible delivery-mode-related redox patterns and supported further investigation of maternal smoking as a potential clinical covariate.

Given the limited sample size, exploratory design, and absence of external validation, these findings should be regarded as hypothesis-generating. Larger external cohorts are needed to confirm the observed patterns, refine the composite indices, and evaluate their possible clinical relevance.

## Figures and Tables

**Table 1:** Descriptive statistics of perinatal biomarkers.

Biomarker/Characteristic	Mothers Mean (SD)	Mothers Median [IQR]	Newborns Mean (SD)	Newborns Median [IQR]
NO_2_^−^ (ng/mL)	18.35 (40.47)	11.80 [7.55–17.02]	17.29 (26.76)	11.54 [7.04–16.38]
NO_3_^−^ (µg/mL)	2.19 (3.57)	1.70 [1.27–2.17]	1.84 (1.04)	1.53 [1.22–2.07]
MDA (ng/mL)	74.36 (94.57)	37.72 [25.22–55.91]	55.83 (59.56)	42.47 [21.50–69.43]
GSH (µg/mL)	3.53 (1.10)	3.38 [2.78–4.22]	3.11 (1.77)	2.59 [2.20–3.21]
GSSG (µg/mL)	2.51 (0.42)	2.52 [2.24–2.76]	1.79 (0.55)	1.75 [1.60–1.87]
GSH/GSSG	1.41 (0.36)	1.35 [1.15–1.58]	1.86 (1.52)	1.53 [1.27–2.04]

**Table 2:** Paired Wilcoxon signed-rank comparison of maternal and neonatal OSI and NSI.

Index	Maternal (Median)	Neonatal (Median)	Wilcoxon Test p-Value	N Pairs
OSI	0.563	−0.350	0.0006	74
NSI	−0.157	−0.311	0.536	74

**Table 3:** Non-parametric composite index correlation results.

Index	Spearman’s ρ	*p*-Value	N Pairs
OSI	−0.128	0.277	74
NSI	0.313	0.007	74

## Data Availability

Data are available upon request and approval from the Ethics Comities of G.E.P. UMPhST and ECCH of Târgu Mures due to privacy restrictions.

## Supplementary Materials

The following supporting information can be downloaded at https://www.mdpi.com/article/10.3390/jcm15156061/s1. Detailed HPLC calibration, analytical standards, reagent preparation protocol P1.

## Abbreviations

The following abbreviations are used in this manuscript:

AUC	Area Under the Curve
CI	Confidence Interval
DNA	Deoxyribonucleic Acid
ECCH	Emergency County Clinic Hospital
GEP UMPhST	George Emil Palade University of Medicine, Pharmacy, Science and Technology of Tirgu Mures
GSH	Reduced Glutathione
GSSG	Oxidized Glutathione
HPLC	High-Performance Liquid Chromatography
IQR	Interquartile Range
NO	Nitric Oxide
NO2	Nitrite
NO3	Nitrate
NO-sGC-cGMP	Nitric Oxide Soluble Guanylyl Cyclase Cyclic Guanosine Monophosphate
NSI	Nitrosative Stress Index
NF-κB	Nuclear Factor Kappa B
MDA	Malondialdehyde
OR	Odds Ratio
OSI	Oxidative Stress Index
p38 MAPK	Mitogen-Activated Protein Kinase 38
RNS	Reactive Nitrogen Species
ROS	Reactive Oxygen Species
SAPK/JNK	Stress-Activated Protein Kinase/c-Jun N-terminal Kinase
SD	Standard Deviation

## References

[1] Turner J.M., Mitchell M.D., Kumar S.S. (2020). The Physiology of Intrapartum Fetal Compromise at Term. Am. J. Obstet. Gynecol..

[2] Cindrova-Davies T., Yung H.W., Johns J., Spasic-Boskovic O., Korolchuk S., Jauniaux E., Burton G.J., Charnock-Jones D.S. (2007). Oxidative Stress, Gene Expression, and Protein Changes Induced in the Human Placenta During Labor. Am. J. Pathol..

[3] Cindrova-Davies T., Spasic-Boskovic O., Jauniaux E., Charnock-Jones D.S., Burton G.J. (2007). Nuclear Factor-Kappa B, P38, and Stress-Activated Protein Kinase Mitogen-Activated Protein Kinase Signaling Pathways Regulate Proinflammatory Cytokines and Apoptosis in Human Placental Explants in Response to Oxidative Stress: Effects of Antioxidant Vitamins. Am. J. Pathol..

[4] Díaz-Castro J., Florido J., Kajarabille N., Prados S., de Paco C., Ocon O., Pulido-Moran M., Ochoa J.J. (2015). A New Approach to Oxidative Stress and Inflammatory Signaling during Labour in Healthy Mothers and Neonates. Oxid. Med. Cell. Longev..

[5] Wu F., Tian F.J., Lin Y. (2015). Oxidative Stress in Placenta: Health and Diseases. BioMed Res. Int..

[6] Poniedziałek-Czajkowska E., Marciniak B., Kimber-Trojnar Z., Leszczyńska-Gorzelak B., Oleszczuk J. (2011). Nitric Oxide in Normal and Preeclamptic Pregnancy. Curr. Pharm. Biotechnol..

[7] Tropea T., Wareing M., Greenwood S.L., Feelisch M., Sibley C.P., Cottrell E.C. (2018). Nitrite Mediated Vasorelaxation in Human Chorionic Plate Vessels Is Enhanced by Hypoxia and Dependent on the NO-sGC-cGMP Pathway. Nitric Oxide.

[8] Mukosera G.T., Principe P., Mata-Greenwood E., Liu T., Schroeder H., Parast M., Blood A.B. (2022). Iron Nitrosyl Complexes Are Formed from Nitrite in the Human Placenta. J. Biol. Chem..

[9] Mukosera G.T., Clark T.C., Ngo L., Liu T., Schroeder H., Power G.G., Yellon S.M., Parast M.M., Blood A.B. (2020). Nitric Oxide Metabolism in the Human Placenta During Aberrant Maternal Inflammation. J. Physiol..

[10] Gitto E., Pellegrino S., Gitto P., Barberi I., Reiter R.J. (2009). Oxidative stress of the newborn in the pre- and postnatal period and the clinical utility of melatonin. J. Pineal Res..

[11] Marseglia L., D’Angelo G., Manti S., Arrigo T., Barberi I., Reiter R.J., Gitto E. (2014). Oxidative Stress-Mediated Aging during the Fetal and Perinatal Periods. Oxid. Med. Cell. Longev..

[12] Perrone S., Negro S., Tataranno M.L., Buonocore G. (2010). Oxidative Stress and Antioxidant Strategies in Newborns. J. Matern. Fetal Neonatal Med..

[13] Aceti A., Beghetti I., Martini S., Faldella G., Corvaglia L. (2018). Oxidative Stress and Necrotizing Enterocolitis: Pathogenetic Mechanisms, Opportunities for Intervention, and Role of Human Milk. Oxid. Med. Cell. Longev..

[14] Buhimschi I.A., Buhimschi C.S., Pupkin M., Weiner C.P. (2003). Beneficial Impact of Term Labor: Nonenzymatic Antioxidant Reserve in the Human Fetus. Am. J. Obstet. Gynecol..

[15] Perrone S., Laschi E., Buonocore G. (2020). Oxidative Stress Biomarkers in the Perinatal Period: Diagnostic and Prognostic Value. Semin. Fetal Neonatal Med..

[16] Perrone S., Laschi E., Buonocore G. (2019). Biomarkers of Oxidative Stress in the Fetus and in the Newborn. Free Radic. Biol. Med..

[17] Grzeszczak K., Łanocha-Arendarczyk N., Malinowski W., Ziętek P., Kosik-Bogacka D. (2023). Oxidative Stress in Pregnancy. Biomolecules.

[18] Griendling K.K., Touyz R.M., Zweier J.L., Dikalov S., Chilian W., Chen Y.R., Harrison D.G., Bhatnagar A., American Heart Association Council on Basic Cardiovascular Sciences (2016). Measurement of Reactive Oxygen Species, Reactive Nitrogen Species, and Redox-Dependent Signaling in the Cardiovascular System: A Scientific Statement from the American Heart Association. Circ. Res..

[19] Marrocco I., Altieri F., Peluso I. (2017). Measurement and Clinical Significance of Biomarkers of Oxidative Stress in Humans. Oxid. Med. Cell. Longev..

[20] Eldridge R.C., Flanders W.D., Bostick R.M., Fedirko V., Gross M., Thyagarajan B., Goodman M. (2017). Using Multiple Biomarkers and Determinants to Obtain a Better Measurement of Oxidative Stress: A Latent Variable Structural Equation Model Approach. Biomarkers.

[21] Katerji M., Filippova M., Duerksen-Hughes P. (2019). Approaches and Methods to Measure Oxidative Stress in Clinical Samples: Research Applications in the Cancer Field. Oxid. Med. Cell. Longev..

[22] Sultana Z., Qiao Y., Maiti K., Smith R. (2023). Involvement of Oxidative Stress in Placental Dysfunction, the Pathophysiology of Fetal Death and Pregnancy Disorders. Reproduction.

[23] Knapen M.F., Peters W.H., Mulder T.P., Merkus H.M.W.M., Jansen J.B.M.J., Steegers E.A.P. (1999). Glutathione and Glutathione-Related Enzymes in Decidua and Placenta of Controls and Women with Pre-Eclampsia. Placenta.

[24] Raijmakers M.T., Bruggeman S.W., Steegers E.A., Peters W.H. (2002). Distribution of Components of the Glutathione Detoxification System Across the Human Placenta After Uncomplicated Vaginal Deliveries. Placenta.

[25] Raijmakers M.T., Steegers E.A., Peters W.H. (2001). Glutathione S-Transferases and Thiol Concentrations in Embryonic and Early Fetal Tissues. Hum. Reprod..

[26] Irestedt L., Lagercrantz H., Hjemdahl P., Hägnevik K., Belfrage P. (1982). Fetal and Maternal Plasma Catecholamine Levels at Elective Cesarean Section Under General or Epidural Anesthesia Versus Vaginal Delivery. Am. J. Obstet. Gynecol..

[27] Eliot R.J., Lam R., Leake R.D., Hobel C.J., Fisher D.A. (1980). Plasma Catecholamine Concentrations in Infants at Birth and During the First 48 Hours of Life. J. Pediatr..

[28] Faxelius G., Lagercrantz H., Yao A. (1984). Sympathoadrenal Activity and Peripheral Blood Flow After Birth: Comparison in Infants Delivered Vaginally and by Cesarean Section. J. Pediatr..

[29] Genc S.O., Erdal H. (2023). Effect of Mode of Delivery on Neonatal Oxidative Stress and Dynamic Thiol-Disulfide Homeostasis. J. Int. Med. Res..

[30] Tok A., Özer S., Özer A., Kurutaş E.B., Baylan F.A., Kılınç M. (2025). The Effect of Mode of Delivery on Oxidative Stress and Antioxidant Parameters in the Mother, Neonate and Maternal Milk. BMC Pregnancy Childbirth.

[31] Karakelleoğlu G., Arslan G., Kırımlı Yanık E.C.N. (2026). Elective Cesarean Preserves Maternal-Fetal Redox Homeostasis, Whereas Emergency Cesarean Disrupts It: A Prospective Observational Study. J. Matern. Fetal Neonatal Med..

[32] Kobayashi H., Iorio E.L., Yoshino A. (2019). Effects of Mode of Delivery on Pro-Oxidant/Antioxidant Balance in Fetal Circulation. J. Matern. Fetal Neonatal Med..

[33] Suter M.A., Aagaard K.M. (2020). The impact of tobacco chemicals and nicotine on placental development. Prenat. Diagn..

[34] Chełchowska M., Gajewska J., Ambroszkiewicz J., Mazur J., Ołtarzewski M., Maciejewski T.M. (2021). Influence of Oxidative Stress Generated by Smoking During Pregnancy on Glutathione Status in Mother-Newborn Pairs. Antioxidants.

[35] Chelchowska M., Ambroszkiewicz J., Gajewska J., Laskowska-Klita T., Leibschang J. (2011). The Effect of Tobacco Smoking During Pregnancy on Plasma Oxidant and Antioxidant Status in Mother and Newborn. Eur. J. Obstet. Gynecol. Reprod. Biol..

[36] Hoch D., Majali-Martinez A., Bankoglu E.E., Stopper H., Glasner A., Desoye G., Gauster M., Hiden U. (2023). Maternal Smoking in the First Trimester and Its Consequence on the Early Placenta. Lab. Investig..

[37] Kawashima A., Koide K., Ventura W., Hori K., Takenaka S., Maruyama D., Matsuoka R., Ichizuka K., Sekizawa A. (2014). Effects of Maternal Smoking on the Placental Expression of Genes Related to Angiogenesis and Apoptosis During the First Trimester. PLoS ONE.

[38] Fogarasi E., Croitoru M.D., Fülöp I., Nemes-Nagy E., Tripon R.G., Simon-Szabo Z., Muntean D.L. (2016). Malondialdehyde levels can be measured in serum and saliva by using a fast HPLC method with visible detection/Determinarea printr-o metodă HPLC-VIS rapidă a concentraţiilor serice şi salivare ale malondialdehidei. Rev. Rom. Med. Lab..

[39] Croitoru M.D. (2012). Nitrite and nitrate can be accurately measured in samples of vegetal and animal origin using an HPLC-UV/VIS technique. J. Chromatogr..

[40] Jîtcă G., Ősz B.E., Tero-Vescan A., Vari C.E. (2021). Psychoactive Drugs-From Chemical Structure to Oxidative Stress Related to Dopaminergic Neurotransmission. A Review. Antioxidants.

[41] Iqbal K., Intemann T., Börnhorst C., Zhang J., Aleksandrova K. (2025). Approaches for harmonization of biomarker data from multiple studies: A narrative methodological review. AJE Adv. Res. Epidemiol..

[42] Skates S.J., Horick N.K., Moy J.M., Minihan A.M., Seiden M.V., Marks J.R., Sluss P., Cramer D.W. (2007). Pooling of Case Specimens to Create Standard Serum Sets for Screening Cancer Biomarkers. Cancer Epidemiol. Biomark. Prev..

