# Supplementary material for: Biomarkers of Maternal and Neonatal Oxidative and Nitrosative Stress During Delivery: An Exploratory Pilot Study on Maternal–Neonatal Adaptation to Stress
Source: J Clin Med. 2026 Aug 4;15(15):6061. doi: 10.3390/jcm15156061 (PMC13467220; doi:10.3390/jcm15156061)
Supplement: Supplementary file 1 [file jcm-15-06061-s001.zip › jcm-4443015-supplementary.pdf]

Supplementary Material:

Detailed HPLC calibration, analytical standards, reagent preparation protocol

Measurement of malondialdehyde

Acetonitrile (600 µL) was added to 200 µL of thawed plasma in Eppendorf tubes, followed by centrifugation at 3000 rpm for 10 minutes. Then, 450 µL of 4 mg/mL thiobarbituric acid (TBA) and 750 µL of 50 mM H_2SO_4 were added to 300 µL of the supernatant. The tubes were vortexed, and the mixture was heated at 100°C for 60 minutes, followed by cooling on ice. Subsequently, the samples were transferred to HPLC vials and analyzed by injection into an HPLC UV/VIS system. The experimental conditions employed were: isocratic elution, a flow rate of 1 mL/min, an injection volume of 100 µL (loop method), and detection at a wavelength of 532 nm; the retention time for MDA was 0.58 minutes.

The chromatographic column used, serving as the stationary phase, was a Supelcosil™ LC-18 (3 µm) SUPELCO 3.3 cm x 4.6 mm column (Merck KGaA, Darmstadt, Germany). The mobile phase consisted of 20 mM phosphate buffer (pH 6) and acetonitrile (VWR International SAS, Fontenay-sous-Bois, France); other reagents included 98% thiobarbituric acid (Sigma-Aldrich, St. Gallen, Switzerland) for MDA derivative formation, 96% sulfuric acid (Chemical Company, Iași, Romania), and a 1,1,3,3-tetramethoxypropane (Sigma-Aldrich, St. Gallen, Switzerland) stock solution for preparing MDA standard solutions. Ultrapure water, obtained via purification, was used to prepare the solutions.

Measurements were made using the Merck-Hitachi HPLC system (Merck KGaA, Darmstadt, Germany) with L-7100 quaternary pump, L-7200 injector, L-7360 column thermostat, a D-7000 interface, an L-7612 solvent degasser, and an L-7455 DAD detector. Other necessary equipment included: a AB54S analytical balance (Mettler-Toledo, Greifensee, Switzerland), a Sigma centrifuge, a T700H ultrasonic bath (Elma Schmidbauer GmbH, Singen, Germany), a Mix 20 vortex mixer (Falc Instruments, Treviglio, BG, Italy), a MP 225 pH meter (Mettler-Toledo Quatro, Greifensee, Switzerland), and a water purification system (Merck KGaA, Darmstadt, Germany).

The sample results were interpreted by comparing the obtained chromatograms with those of a series of standard solutions containing known concentrations of MDA, ranging 0.52-262 ng/mL [38].

Measurement of nitrate and nitrite

The mobile phase used for nitrate and nitrite measurement was 5 mM tetrabutylammonium hydroxide adjusted to pH 2.5 with sulfuric acid, acetonitrile, and methanol; injection volume was 100 µl; maximum absorbance was obtained at 222 nm for nitrate and at 520 nm for derivatized nitrite. Reagents: Griess Reagent A: 50 mg of sulphanilic acid was dissolved by ultrasonication in a mixture of 1.5 ml acetic acid and 3 ml purified water. Griess Reagent B: 4 mg of 1-naphthylamine was dissolved in 4 ml acetic acid. After preparation, the Griess A and Griess B solutions were stored in the refrigerator and used within 3 days. All the reagents for this procedure were purchased from Merck KGaA (Darmstadt, Germany).

After thawing, 200 µl of 5 mM tetrabutylammonium hydroxide (adjusted to pH 2.5) was added to 200 µl of supernatant; following homogenization, the mixture was centrifuged at 8,000 rpm for 10 minutes. A 150 µl aliquot of the resulting supernatant was pipetted into an HPLC vial, and 15 µl of

Griess Reagent A was added; after homogenization, an equal volume (15 µl) of Griess Reagent B was added. Following vigorous homogenization, the sample was stored in a refrigerator until analysis.

The blank was prepared using 200 µl of purified water, to which 200 µl of acetonitrile and 400 µl of TBA (thiobarbituric acid) were sequentially added; the solution was transferred to an HPLC vial, followed by the sequential addition of 80 µl each of Griess Reagent A and Griess Reagent B, and vigorous homogenization. The ion-pair technique was used in conjunction with reversed-phase chromatography, allowing for the simultaneous determination of nitrate and nitrite (via UV detection for nitrate and visible detection for derivatized nitrite). The determinations were performed using the HPLC-UV/VIS technique on an equipment from Merck KGaA (Darmstadt, Germany) with the following configuration: Merck Hitachi L-7100 quaternary pump, Merck Hitachi L-7200 autosampler, Merck Hitachi L-7360 column thermostat, Merck Hitachi L-7455 DAD detector, Merck Hitachi L-7000 interface, Merck Hitachi L-7612 solvent degasser, D-7000 HSM-Manager software, LichroCART 250-4, Lichrospher 100, RP-18 (5 µm) Merck KGaA column, and LichroCART 4-4, Lichrospher 100, RP-18 (5 µm) Merck KGaA guard column. Additionally, LCMS-QP 8000 mass detector (Shimadzu Corporation, Kyoto, Japan); XBridge C18, 2.5 µm, 2.1 mm x 50 mm column (Waters Corporation, Saint-Quentin-en-Yvelines, France) were used.

The calibration curve was obtained by injecting standard solutions with known concentrations of nitrate (0.2–40 µg/mL) and nitrite (10–400 ng/mL) into the HPLC system; these standards underwent the same preparation steps as the samples [39].

Measurement of oxidized and reduced glutathione

To determine reduced glutathione levels after thawing, 150 µL of trichloroacetic acid was added to the samples, followed by centrifugation at 10000 rpm for 20 minutes; the supernatant was then transferred to HPLC vials and stored until the day of analysis. For GSSG determination, samples were heated to 80°C for 30 minutes prior to the addition of trichloroacetic acid — a step that induced reduction of glutathione — after which the mixture was cooled, trichloroacetic acid was added, and the samples were centrifuged at 10000 rpm for 20 minutes. The supernatant was transferred to HPLC vials and stored until analysis. Standard solutions for GSH and GSSG were prepared using similar procedures. Ellman's reagent can react with GSH to form a GSH-DTNB dimer. Quantitative determination of GSSG is performed by calculating the difference between total glutathione and reduced glutathione. Total glutathione can be determined by reducing oxidized glutathione to reduced glutathione.

The instrument used was a Merck-Hitachi UV/VIS HPLC system (Merck KGaA, Darmstadt, Germany), comprising the following components: an L-7100 quaternary pump, an L-7200 injector, an L-7360 column thermostat, a D-7000 interface, an L-7612 solvent degasser, and an L-7455 DAD detector. Other equipment used included: an analytical balance (Mettler-Toledo AB54S), a centrifuge from Sigma-Aldrich (St. Gallen, Switzerland), a T700H ultrasonic bath (Elma Transsonic GmbH, Singen, Germany), a Mix 20 vortex mixer (Falc Instruments, Treviglio, BG, Italy), a MP 225 pH meter (Mettler-Toledo Quatro, Greifensee, Switzerland), and a water purification system (Merck KGaA, Darmstadt, Germany).

The chromatographic column used as the stationary phase was a Zorbax Eclipse XDB-C8 (5 µm, 4.6 x 150 mm) from Agilent Technologies (Santa Clara, CA, USA); the mobile phase consisted of phosphate buffer (20 mM, pH 2.5) and acetonitrile (VWR International SAS, Fontenay-sous-Bois, France). Anhydrous Na_2HPO_4 and 85% H_3PO_4 (both from Merck KGaA, Darmstadt, Germany) were used to

prepare the phosphate buffer; these reagents were also required to prepare the dissolution solution for Ellman's reagent (reaction buffer, pH 8). Additional materials included reduced and oxidized glutathione standards (Sigma Aldrich, St. Gallen, Switzerland), 5,5'-dithiobis (2-nitrobenzoic acid)—i.e., Ellman's reagent (Sigma Aldrich, St. Gallen, Switzerland), —trichloroacetic acid (Chemical Company, Iași, Romania) for protein precipitation, and ultrapure water for solution preparation.

The experimental conditions used for the analysis were as follows: flow rate of 1 mL/min, injection volume of 50 µL using the loop method, detection wavelength of 330 nm, and a retention time of 9.11 ± 0.15 (SD) minutes for the GSH-DTNB dimer. The sample results were interpreted by comparing the obtained chromatograms with those of a series of standard solutions (ranging 0.5-100 µg/mL) containing known concentrations of GSH and GSSG, respectively [40].

Following the analysis of the decomposition of Ellman's reagent at 80 °C, no interferences were observed with the retention time of the analyte of interest. In the case of plasma, no peaks overlapping the signal given by GSH-DTNB were observed either.

The percentage of reduction of oxidized glutathione is an important factor in the applicability of the method. The calculation of the percentage of reduction was performed by comparing the area under the curve of water or serum samples with the same amount of GSSG or GSH. The reduction percentage was verified at 5 concentration levels with three series of samples. The reduction efficiency was 97.11 ± 6.13 (SD) % in plasma samples.

In the presented method of glutathione measurement, the lower limit of detection (LOD) was 0.04 µg/mL and the LOQ (lower limit of quantification) was 0.14 µg/mL. The coefficient of variation at the LOD concentration was 7.79 % based on 3 series of 5 standard solutions.

The linearity of the method was verified for both reduced and oxidized glutathione in water and plasma as matrices in the 0.5-100 µg/mL concentration range showing a linear pattern with a correlation coefficient exceeding 0.99.

The accuracy of the method was verified at three concentration levels: 10, 30, 70 µg/mL, with 5 sample series each, from water and plasma matrices. The accuracy of the method was expressed as the percentage recovery of the concentration calculated based on the equation of the straight line against the theoretical concentration, based on the equation obtained from the calibration curve. The average values were within the recovery range of 85-115 %.

The precision of the method was characterized using the coefficient of variation, and the calculated values fell within the limits allowed in the bioanalytical validations (below 15%), the coefficient of variation fell between the values of 0.78-5.68% and the percentage of accuracy between the range of 90.89-105.02%.

The recovery of analytes (GSH, GSSG) was calculated at 4 concentration levels with three series each by comparing the area of the peaks determined from plasma samples to the area of the peaks obtained from water. In the absence of a blank (plasma that does not contain glutathione), in the first phase the peak area corresponding to endogenous glutathione in plasma was determined. By subtracting this value from the areas determined for samples with different amounts of reduced or oxidized glutathione, and comparing them with the values obtained from water, the percentage recovery of the analyte was determined. The total recovery of reduced glutathione was 100.59 ± 12.83 (SD) and that of GSSG as reduced glutathione was 79.13 ± 6.99 (SD) in plasma samples.

Data regarding the validation of the GSH/GSSG methodology were described based on the PhD thesis entitled: Research on the phytochemistry and some biological effects of red wine, authored by Erzsébet Májai (Fogarasi) in 2018.
